# Sensitivity Analysis of a Portable Wireless PCB-MEMS Permittivity Sensor Node for Non-Invasive Liquid Recognition

**DOI:** 10.3390/mi12091068

**Published:** 2021-09-02

**Authors:** Javier Meléndez-Campos, Matias Vázquez-Piñón, Sergio Camacho-Leon

**Affiliations:** Tecnologico de Monterrey, School of Engineering and Sciences, Ave. Eugenio Garza Sada 2501, Monterrey 64849, Mexico; A00819577@itesm.mx (J.M.-C.); matias.vazquez@tec.mx (M.V.-P.)

**Keywords:** PCB MEMS, permittivity sensor, liquid recognition

## Abstract

Dielectric characteristics are useful to determine crucial properties of liquids and to differentiate between liquid samples with similar physical characteristics. Liquid recognition has found applications in a broad variety of fields, including healthcare, food science, and quality inspection, among others. This work demonstrates the fabrication, instrumentation, and functionality of a portable wireless sensor node for the permittivity measurement of liquids that require characterization and differentiation. The node incorporates an interdigitated microelectrode array as a transducer and a microcontroller unit with radio communication electronics for data processing and transmission, which enable a wide variety of stand-alone applications. A laser-ablation-based microfabrication technique is applied to fabricate the microelectromechanical systems (MEMS) transducer on a printed circuit board (PCB) substrate. The surface of the transducer is covered with a thin layer of SU-8 polymer by spin coating, which prevents it from direct contact with the Cu electrodes and the liquid sample. This helps to enhance durability, avoid electrode corrosion and contamination of the liquid sample, and to prevent undesirable electrochemical reactions to arise. The transducer’s impedance was modeled as a Randles cell, having resistive and reactive components determined analytically using a square wave as stimuli, and a resistor as a current-to-voltage converter. To characterize the node sensitivity under different conditions, three different transducer designs were fabricated and tested for four different fluids, i.e., air, isopropanol, glycerin, and distilled water—achieving a sensitivity of 1.6965 +/− 0.2028 ε_r_/pF. The use of laser ablation allowed the reduction of the transducer footprint while maintaining its sensitivity within an adequate value for the targeted applications.

## 1. Introduction

Dielectric characteristics are useful to determine the crucial properties of liquids and to differentiate between liquid samples with similar physical characteristics. Liquid recognition has found applications in a broad variety of fields, including healthcare [1], food science [2], and quality inspection [3], among others. The capabilities of sensors for liquid characterization have been studied before, using interdigitated electrodes arrays (IDEA) in applications that require the differentiation of liquids in certain environments in which conditions must be controlled. In this regard, one of the most used parameters to assess liquids is the dielectric constant. Due to its capacitive nature, IDEAs are suitable for measuring dielectric changes across their electrode fingers.

Even though the data generated by one sensor could gather enough information for some applications, the conjunction of several sensors as a network is what actually manifests the potential of the Internet of Things (IoT) paradigm. Each sensor in a spatially distributed network is called a sensor node and is hooked up to a network by a communication protocol, such as Wi-Fi, MQTT, Bluetooth Low Energy (BLE), among others.

Recent technological advances on Micro-Electro-Mechanical Systems (MEMS) have allowed the development of cost-effective, portable, and low power-consuming sensors nodes that integrate sensing, processing, storage, and communication capabilities in a single device [4]. These devices merge electronic, mechanical, and even microfluidic systems in the same die. A sensor node in a Wireless Sensor Network (WSN) is an accurate, autonomous, small, and affordable system, which usually integrates the sensing device, i.e., a transducer that converts energy in the form of a targeted physical, chemical, or biological signal to an output energy that is readable by a measuring mechanism—, power supply, radio and data transceivers, measuring circuits, and a main microcontroller.

To achieve granularity and heterogeneity on the information that a sensor network could generate, it must include as many nodes as possible. MEMS technology has allowed the development of low-cost sensors that open the possibility of large-scale deployment and scalability for WSN. As part of a system, the transducer works as the input element for the sensor node, i.e., it measures a defined physical, chemical, or biological variable, and generates associated quantitative data. Since the sensor nodes are deployed in different environments, the transducer must maintain a small form factor and must be capable of withstanding the environmental conditions under which it is exposed to. However, transducer miniaturization involves high resolution fabrication processes to achieve small feature sizes that allows the maximization sensitivity without compromising the overall footprint of the device. In this sense, MEMS fabrication processes, such as printed circuit board (PCB)-MEMS, allows a tradeoff to be found between high-resolution geometries and small form factors for the design of high-sensitivity transducers.

The resolution and range of the data generated by the nodes also depends on the instrumentation circuitry, which filters, transforms, and manages signals from the transducer and reports a bounded, punctual output signal for analysis to the microcontroller. The instrumentation stage is generally composed by either passive or active elements. Complex instrumentation circuits may increase the overall range and resolution of the transducer; however, this involves an important power usage or a significant size increase of the system. The communication interface transmits the signals in a defined standard protocol to a remote device. The size and latency of the reported data directly impact the reliability and independence of the sensing node, hence the need to optimize the report the intervals and package sizes of the communication block. The general approach is to send representative data only when it is needed.

The accessibility to sensor nodes is not always feasible once deployed; hence, power efficiency is considered a design priority in order to maximize the long-term autonomy of the device. In this sense, batteries and energy harvesting techniques are the most common types of power source used in WSN.

Although the concurrent optimization of all subsystems is challenging for a given design, each application of wireless sensing nodes dictates a subset of characteristics to be optimized with a corresponding tradeoff involved.

Furthermore, multiple instrumentation circuits have been implemented to measure and characterize physical variables in sensing nodes. These circuits normally involve the use of external integrated circuits to detect changes in the transducer output signal to later send the signals to a microcontroller for analysis [5,6]. Additionally, some other implementations have demonstrated the detection of changes in physical variables using complex data processing algorithms, such as neural networks [7]. Regarding power consumption, on the one hand, optimization techniques in battery-powered devices have been explored in order to extend the node longevity on the field [8,9]. On the other hand, the use of energy harvesting techniques is increasingly common due to their ability to gather data without the use of batteries. This requires energy-aware approaches where the data rates and size are optimized [10].

The improvement of WSN communications is also fundamental when working with multiple sensor nodes. One example of such efforts is the study of real-time methods for link quality estimation for the industrial applications of wireless sensor nodes [11]. The applications of WSN and their nodes have been widely analyzed in areas such as agriculture [12,13,14], logistics [15], industry [11,16], military defense [17], among many others.

Regarding liquid recognition, different platforms have been developed, integrating sensing and functionalization techniques for general-purpose applications. For example, the use of paper microfluidics incorporated in a wireless sensor node that employs RFID technology to integrate a disposable, lightweight, and flexible device with a permittivity sensing of 15%/ε_r_ [18]. Alongside this, other platforms have been developed for the use of screen-printing technology on an alumina substrate to detect linear pH changes on a RuO2 sensitive layer using conductimetric analysis [19]. Finally, two different sensors nodes based on the Substrate-Integrated-Waveguide (SIW) resonant cavity have been developed and tested using a frequency analysis with six different liquids, achieving a sensitivity of 1.03 MHz/ε [20].

Even though silicon has been used as substrate in most systems involving transducers due to its physical and electrical properties [21,22], the most commonly used silicon micromachining technique is photolithography, which is employed in the integrated circuit industry and requires specific infrastructure, such as cleanrooms with very high-quality standards and is normally found only in highly specialized companies or research centers.

Alternative technologies have been explored since the 1990s to allow the rapid prototyping of MEMS at a low cost and without the use of specialized tools or facilities at the expense of geometry resolution. Accordingly, PCB-MEMS use regular PCB as a substrate and allows maskless techniques, such as laser ablation and milling, to transfer previously designed patterns to the PCB in a faster development cycle and achieving a resolution as low as 50 µm [23,24]. PCB fabrication techniques have been previously used in non-invasive liquid recognition applications using IDEAs, demonstrating the feasibility of this process in the field of liquid sensing [25].

In this work, the maskless fabrication technique using laser ablation to develop PCB-MEMS devices is explored to fabricate an IDEA-based impedance transducer for liquid recognition. The IDEA is integrated into a wireless sensor node that implements microcontroller for data processing and signal generation, a BLE module for data transmission, and an external power source. The microcontroller implements an algorithm to calculate the transducer impedance from a three-voltage measurement technique that was previously reported [26], and we take advantage of the programmable system-on-chip (PSoC) capabilities to integrate signal generation, data reading, and data transmission on a single microcontroller unit (MCU), which enables full IoT functionality for a wide variety of stand-alone applications. One possible use of this sensor node is in industrial applications to detect the presence of water and pollutants in oil-powered machines in order to prevent damages. Additionally, the sensor can gather data in environmental sensing networks in order to approximate the medium conditions in open environments.

## 2. Materials and Methods

### 2.1. Transducer

**Figure 1:**
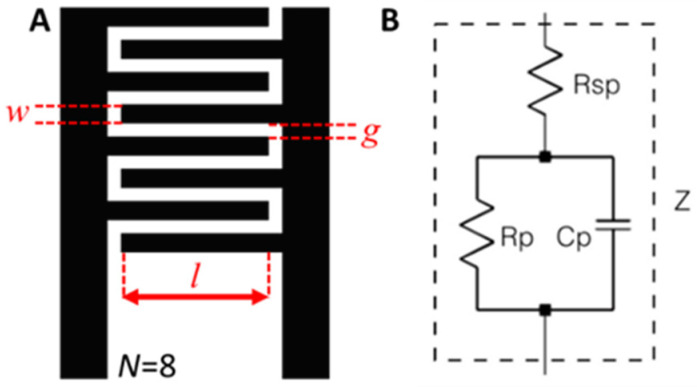
Design parameters of the IDEA: (**A**) geometrical design variables for the IDEAs, and (**B**) equivalent circuit of the IDEA using a three-component circuit.

The IDEA transducer described in this paper is considered to be a symmetric coplanar. Furthermore, the surface of the transducer is covered with a thin layer of SU-8, which prevents direct contact between the metallic electrodes and the liquid sample. This helps to enhance durability, avoid electrode corrosion and contamination of the liquid sample, and also helps to prevent undesirable electrochemical reactions from arising. For this reason, the transducer is considered to be non-invasive to the sample volume. Figure 1A shows the geometric variables of the IDEA, which are the finger count (N), the gap between adjacent fingers (g), finger width (w), and the finger overlapping length (l). Figure 1B shows the equivalent circuit of the electrodes and their interface with the medium under test [27]. The equivalent circuit incorporates the medium resistance in series with the electrode’s resistance, simplified into $R_{sp}$. The model also includes a double layer capacitance due to the charge transfer process (Cp) at the electrode–electrolyte interface and a charge transfer or polarization resistance (Rp).

The double layer capacitance between the metal electrodes and the liquid sample in the upper half-space can be calculated by [28]

(1) $$C_{l}=\frac{\varepsilon_{0}\varepsilon_{r}}{2}\frac{K\left(\sqrt{1-q^{2}}\right)}{Κ\left(q\right)},$$

where $\varepsilon_{0}=8.854\times 10^{-12}\;F\cdot m^{-1}$ is the vacuum permittivity, $\varepsilon_{r}$ is the dielectric constant of the liquid sample, and Κ(q) is the complete elliptic integral of the first kind, as determined by [28]

(2) $$Κ\left(q\right)=\int_{t=0}^{1}\frac{dt}{\sqrt{\left(1-t^{2}\right)\left(1-q^{2}t^{2}\right)}}.$$

Here, q is a geometric term that depends on the electrode width, w, and the gap between adjacent electrodes, g. For a parallel plate capacitor, it can be calculated by [28]

(3) $$q=\frac{g}{g+w}.$$

Meanwhile, for multiple plates in parallel, it is calculated using [28]

(4) $$q=\cos\left(\frac{\pi }{2}\frac{w}{g+w}\right).$$

Since the total parallel fingers integrated in an IDEA is represented by N, the total capacitance of the electrode is the sum of all of the parallel plates in it (N−1) and can be approximated by [28]

(5) $$C_{p}=C_{l}\left(N-1\right)l.$$

The remaining substrate capacitance in the lower half-space is determined experimentally by a linearity analysis and a calibration stage, detailed in Section 3.4. Experimental tests in which by subtracting an offset capacitance from the total signal, the results presented only contain information from the upper half-plane (medium under testing).

### 2.2. Fabrication Process

As seen in the previous section, the dimensions of IDEA transducers directly impact its measurement range and sensitivity. As the transducer footprint size increases, the sensitivity is improved. Meanwhile, as the size of gaps between the interdigitated fingers are reduced, the sensitivity is also increased. The miniaturization of these IDEA transducers then demands fabrication techniques that could achieve well defined electrode fingers with gaps as small as possible between adjacent fingers. Laser ablation is a cost-effective technique that allows the fabrication of well-defined structures and small gaps in a small footprint area. This technique overcomes the lack of precision of traditional PCB machining techniques, while reducing the fabrication time and cost, compared to photolithography, at the prototyping stage. Hence, laser ablation allows the rapid prototyping of small-form sensor nodes while reducing the steps of the development cycle.

**Figure 2:**
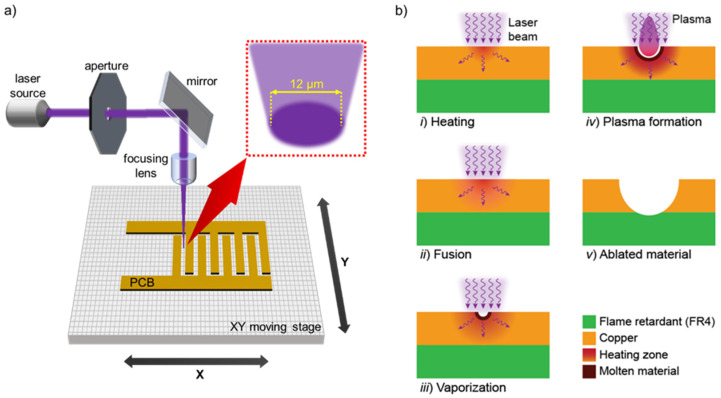
Conventional laser ablation process for PCB-MEMS fabrication; (**a**) schematic diagram of the optical setup and (**b**) the material removal steps by laser irradiation.

To fabricate the transducers, the laser ablation process on a PCB described in Figure 2 was followed. Figure 2a shows the schematic diagram of the optical setup, where a pulsating laser beam is passed through an aperture and is reflected on a mirror array to finally be focused on the PCB surface where ablation takes place. In this case, the laser source is static, and the stage where the PCB is resting moves in the XY plane. Figure 2b depicts the stages of the laser ablation process. Here, the pulsating laser beam is used to ablate the solid material from a substrate at the focused area by (i) heating the material surface, which leads to (ii) material fusion and (iii) vaporization. Eventually, the material transforms to the (iv) plasma state and is released to the ambient space. The (v) ablated material is mainly the copper layer of the PCB even though a small portion of the substrate (FR4) is removed too. The ablation processing unit used in this work (LPKF ProtoLaser U3, LPKF Laser & Electronics, Garbsen, Germany) comprises a Nd:YAG laser operating at a wavelength of 355 nm, with a transversal intensity characterized by a Gaussian profile. The focused laser beam has a diameter of 12 µm. The laser was configured with an average power of 4.49 W and a pulse frequency of 53.75 kHz. The PCB consists of a 1.6 mm-thick FR4 substrate covered by an 18 µm-thick Cu layer on one of the planar surfaces. The IDEA pattern was designed in a Computer Aided Design (CAD) software and was then loaded on the ablation processing unit to transfer the pattern to the Cu layer of the PCB.

A total of three different IDEA designs were fabricated in order to compare the effect of different effective footprint areas on the resulting capacitance Cp. Table 1 shows the geometrical characteristics of the fabricated prototypes. As IDEAs have been built from the same substrate, the thickness (h) of all of their fingers is 18 µm. The rationale for determining the dimensions (w, g, l) and finger count (N) is to explore the design space by keeping the sensor footprint area (A) as invariant as possible, first, by only varying the number of fingers from 20 to 24 (IDEA1 vs. IDEA2) and then by varying their length from 4.3 mm to 5.2 mm (IDEA1 vs. IDEA3).

**Figure 3:**
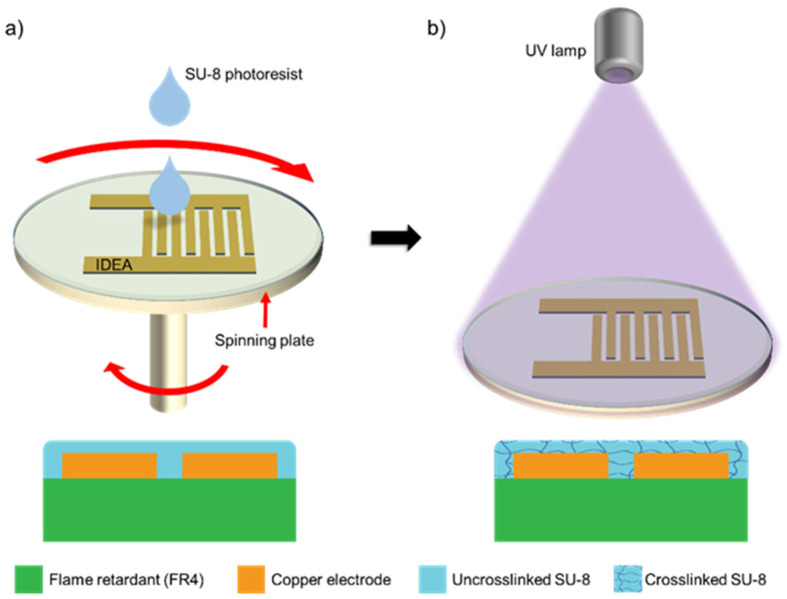
SU-8 deposition process on the PCB: (**a**) spin-coating of PCB with uncrosslinked SU-8 mixed with solvents to a thickness of 2 µm and (**b**) UV exposure of SU-8 for crosslinking after a soft-bake process to remove solvents.

Once the IDEA patterns were transferred to the PCB, post-processing was conducted to deposit the SU-8 layer over the Cu structures. Here, the IDEA surface was spin-coated with a 2 µm-thick layer of SU-8 photoresist (Figure 3a) and was exposed to UV light for crosslinking after a soft-bake to accelerate the polymerization process (Figure 3b). The SU-8 layer works as a protective layer to the Cu structures to avoid corrosion and sample contamination in order to prolong the operational lifetime of the transducer.

### 2.3. Finite Element Analysis

**Figure 4:**
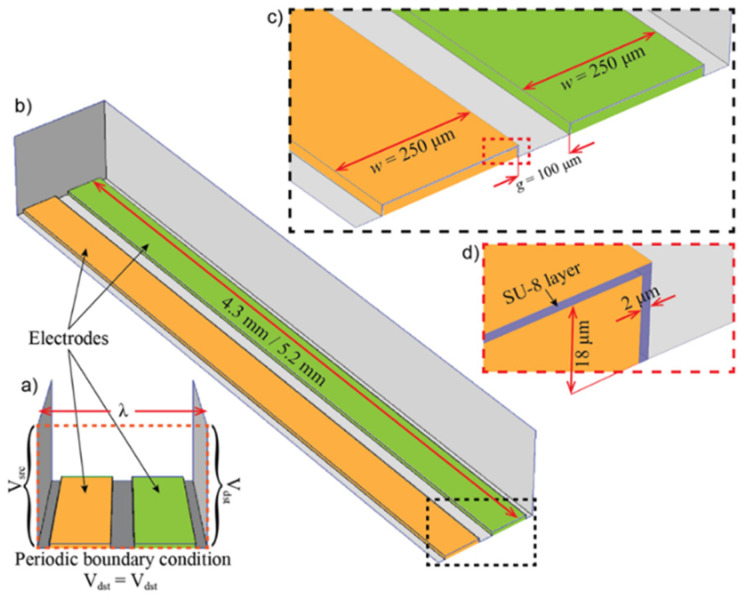
3D geometric definitions and established boundary conditions for the finite element analysis: (**a**) periodic section of IDEA; (**b**) full 3D geometric domain, where l = 4.3 mm for IDEAs 1 and 2 and l = 5.2 mm for IDEA 3; (**c**) inset showing electrode width and a gap between adjacent electrodes; and (**d**) inset showing electrode thickness and SU-8 layer thickness.

A 3D model of a short section of the IDEA was used in a finite element analysis with COMSOL Multiphysics (COMSOL Inc., Stockholm, SE) to numerically study the electric field distribution, ***E***, around the electrode vicinity of the designed structures by means of Gauss’ Law. The 3D geometry includes a periodic section of one electrode pair forming the IDEA, defined as λ=2(w+g), as shown in Figure 4a. The full geometry designed for the analysis is shown in Figure 4b. Here, IDEA 1 and IDEA 2 have an electrode length of l = 4.3 mm, and IDEA 3 has an electrode length of l = 5.2 mm. Figure 4c shows the electrode width, w, and the gap between electrodes, g (omitting the SU-8 layer’s thickness). Finally, Figure 4d shows the electrode thickness, h, and the thin SU-8 layer covering the electrodes.

The resulting mesh for 4.3 mm long electrodes (IDEA 1 and 2) is composed of 261,085 domain elements. For the case of IDEA 3,the electrodes were 5.2 mm long, and the mesh was composed of 314,833 domain elements. In all cases, the meshes were designed to have a minimum element size of 17.2 µm at the electrodes surface and a growth rate of 1.4. The maximum element size was determined to be around 100 µm at the farthest locations from the electrodes, where |***E***| is assumed to be practically zero. The analysis considers that (a) the problem is electrostatic due to the very low frequencies involved, (b) the electric potential is given by the Laplace equation with suitable conditions in the metal–dielectric and dielectric–dielectric boundaries, and (c) the electric field, ***E***, is obtained as the negative of the electric potential gradient

(6) E=−∇V.

### 2.4. Experimental Setup

**Figure 5:**
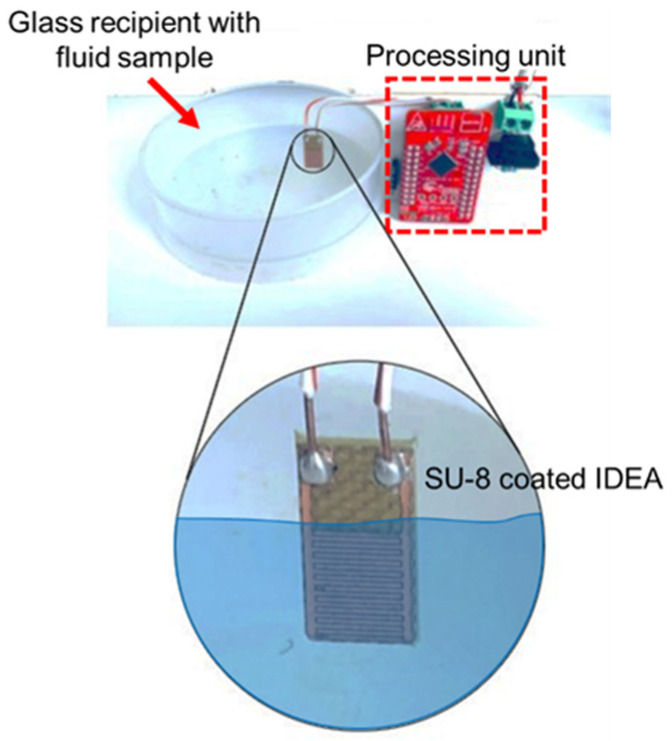
Experimental setup.

In order to validate the performance of the sensor node, four different fluid samples were characterized, i.e., air, isopropyl alcohol, glycerin, and distilled water. These fluid samples were chosen due to their widespread dielectric constants: $\varepsilon_{r}$ = 1 for air, $\varepsilon_{r}$ = 17.9 for isopropyl alcohol, $\varepsilon_{r}$ = 45 for glycerin, and $\varepsilon_{r}$ = 80 for distilled water. Different 60 mL glass recipients were used to test the transducer performance for each fluid sample and all of the tests were conducted at room temperature. The transducer was vertically immersed in the testing fluid, exposing only the SU-8-covered metallic surfaces to the sample and ensuring that the solder pads did not reach the fluid, as shown in Figure 5. Measurements were taken after 2 min intervals, and then the transducer was removed from the fluid sample. All of the transducers were cleaned using isopropanol and distilled water after each measurement.

**Figure 6:**
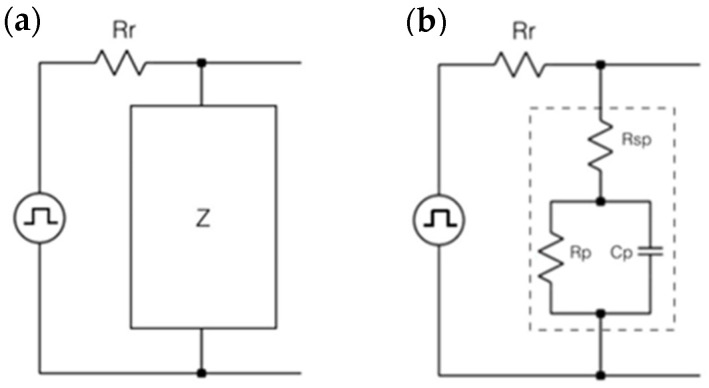
Sensor node structure with current-to-voltage converter (**a**) connected in series to the impedance transducer (**b**) where the relation between the capacitance and the properties of the liquid under test.

The Randles cell was used to model the electrical behavior of the IDEA, which consists of a resistor $R_{r}$ (proposed with a value of $R_{r}$ = 1 MΩ to achieve capacitances in order of pF) working as a Current-to-Voltage-Converter (CTVC) connected in series to the impedance transducer (Z), as shown in Figure 6a. The principle of operation is a voltage divider. The Randles cell is formed by a polarization resistance, $R_{p}$, connected in parallel with the Electric Double Layer capacitance—the capacitance at the interface between the electrode surface and the electrolyte—$C_{p}$ [28,29], and this, in series with the electrolyte resistance, $R_{sp}$. Figure 6b shows the Randles cell equivalent circuit of the impedance transducer, where the relation between the capacitance and the properties of the liquid under test is given by Equations (1)–(5).

The circuit is stimulated by a square wave pulse with a period $T_{p}$ = 1 ms and a 50% duty cycle generated by the microcontroller. The capacitance range of $C_{p}$ is assumed to be in the order of the picofarads in order to determine values for $R_{r}$ and $T_{p}$. Based on three different voltage measurements at $V_{out}$, the value of the components that conform to Z ($R_{sp}$, $R_{p}$, $C_{p}$) can be analytically deduced as in [26].

### 2.5. System Integration

**Figure 7:**
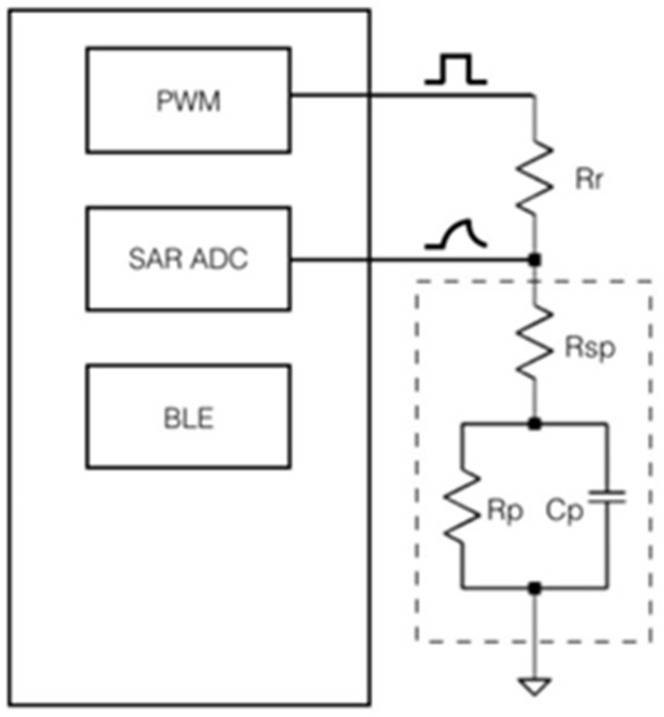
Sensor node schematic diagram.

The reported system is a sensor node for wireless sensing networks that satisfy the requirements to be implemented as a part of an IoT application. A complete scheme of the system is shown in Figure 7. The system detects changes of impedance caused by variations of $R_{sp}$, $R_{p}$, and $C_{p}$, which correspond to the IDEA interactions with the testing fluid. Using the CTVC and an analytical algorithm, the microcontroller calculates the value of each element of the decomposed transducer (Z) by sensing $V_{out}$ at three different times, as described in [26]. Certainly, more points could be considered to improve the quality of the measure, but it would imply making the processing stage more complex, thus increasing its demand for resources (power, time) and compromising its energy autonomy. Finally, the data is sent over BLE for further analysis.

The low-cost 32-bit, 48 MHz CY8C424LQI PSoC (Cypress Semiconductors Corporation, San Jose, CA, USA) was implemented as the MCU for the designed sensor node. This MCU is embedded into the CY8CKIT-042-BLE testing board, which exposes all of the input/output pins for the general use of the microcontroller. The timer-counter-pulse width modulation (TCPWM) module is used to generate the square-voltage pulse in one of the microcontroller outputs. The analog-to-digital-converter (ADC) integrated in the PSoC is a Successive-Approximation Register ADC (SAR ADC) and is used to measure $V_{out}$ at three different time points during the duty cycle of $T_{p}$, as required by the Z component calculation algorithm implemented to determine the values of $R_{sp}$, $R_{p}$, and $C_{p}$, following a previously reported procedure [26].

**Figure 8:**
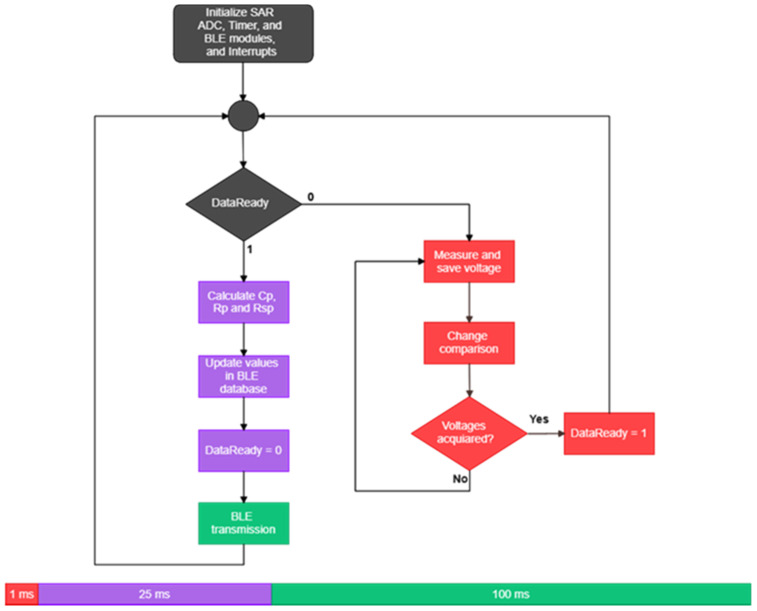
Flow diagram of the algorithm implemented in the PSoC module. The timeline diagram shows the estimated timing for each process of the algorithm, including voltage measurements $V_{k}$, $V_{l}$, and $V_{m}$, calculation of $R_{sp}$, $R_{p}$ and $C_{p}$, and wireless data transmission over BLE.

The flow diagram of the calculation algorithm implemented in the MCU is shown in Figure 8. Briefly, all of the subsystems and interruptions are properly set and initialized. Then, the timer—with a frequency of 2 MHz—is set to provide a signal with a period of 1000 clock pulses, and a Compare field set to $t_{k}=T/8$ is started. When the timer reaches $t_{k}$, an interruption routine is fired, and the ADC performs the voltage measurement $V_{k}$. The Compare field is then set to $t_{l}=T/2$, and the measuring procedure is repeated by the ADC to obtain $V_{l}$. The next measurement is taken at $t_{m}=7T/8$, and the DataReady flag is set to 1. Finally, the value calculation algorithm is computed in the main loop to determine the value of the three Z components. The data are then updated in the Generic Attribute Profile (GATT) database of the BLE module, and the clients subscribed to notifications for certain characteristic are informed of the change of value. Based on the algorithm processing times and the BLE transfer rate, the total process takes approximately $126T_{p}$.

A BLE custom profile called the Impedance Profile was programmed in the PSoC. Furthermore, a custom service named SPIC containing the information for the calculations of $R_{sp}$, $R_{p}$, and $C_{p}$ service was also programmed. The sensing node acts as a Generic Access Profile (GAP) server in order to provide information about its characteristics to the appropriate clients.

In order to establish a link with the Sensing Node, the BLE client (usually a gateway) must scan for Impedance Profile devices. Once a device with such characteristics is detected, the client asks for a connection to start. The sensor node then initiates the connection and exposes all of its characteristics. The BLE client might read the characteristics or can subscribe to them; this way, the sensing node can notify the client when the characteristics have changed.

## 3. Results and Discussion

### 3.1. Transducer Fabrication

**Figure 9:**
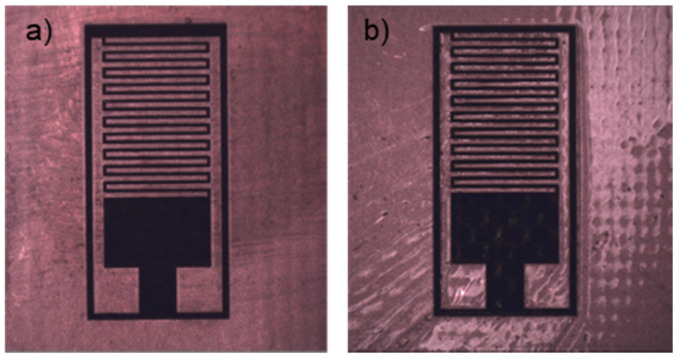
Fabricated IDEAs: (**a**) before and (**b**) after SU-8 deposition.

A geometrical analysis of the fabricated structures, shown in Figure 9, provides a reliable evaluation of the ablation process followed to transfer patterns to the PCB, particularly for characteristic design features of the IDEA design, such as electrode width, length, and gap between electrodes. In this sense, a comparison between geometrical features between the ideal CAD design and the ablated PCB-MEMS structure of one of the devices would serve as a reference to characterize the aforementioned process followed in this work. For this, a device with N = 20 was used, and 20 samples of each geometrical characteristic, i.e., w, l, and g —were taken from the ablated device using an image processing software (ImageJ, National Institute of Health, Bethesda, MD, USA); the means and standard deviations (SD) are presented and contrasted to the ideal feature sizes from the CAD design. Table 2 presents the characterization results.

Even though the focused laser beam features a 2 µm diameter, the overall laser ablation system resolution shows a small relative percentage error for feature sizes larger than 100 µm. This might be caused by a low scanning frequency of the XY moving stage, combined with a high laser beam power for the ablated material’s composition and thickness. Moreover, as previously shown in Figure 2b, material removal depth is not completely isotropic due to the isothermal lines correlated to the Gaussian distribution of the laser beam intensity or to the intrinsic absorption properties of the material [24]. This characterization results suggests that a fine-tunning step is required for applications where accurate pattern transfer is critical to keep RPE as low as possible. For this work, considering an 18 µm-thick Cu layer, feature sizes of the characteristic parameters are as large as 10.8% at the Cu layer surface, i.e., the plane where the characterization was carried out. At the bottom of the Cu layer, feature sizes are assumed to be smaller; however, this is not characterized in the current work.

### 3.2. Capacitance Simulation

**Figure 10:**
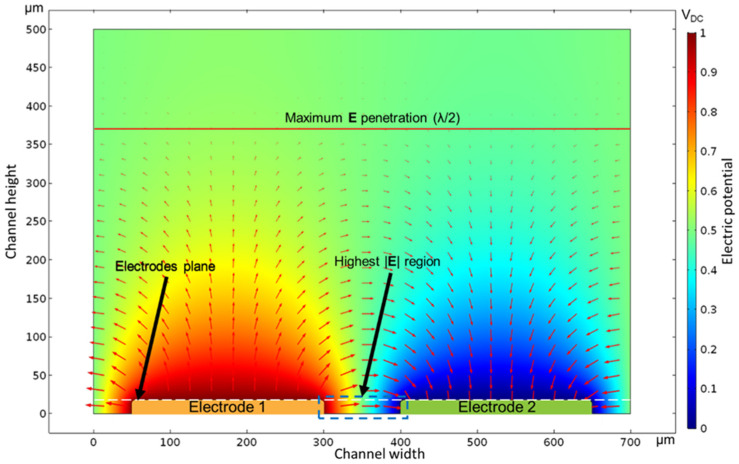
Electric potential and electric field distribution of a XY plane cut from the 3D model from the finite element analysis. Color gradients correspond to the potential electric distribution across the testing liquid. A potential differential of 1 VDC is applied to Electrode 1 with respect to Electrode 2. Arrows represent the electric field distribution, E=−∇V.

The computation of the 3D representation of a λ section of the transducer results in the electric potential distribution across the testing fluid, as shown in Figure 10. Here, a potential of 1 VDC is applied to Electrode 1, taking Electrode 2 as the reference. Red arrows represent the electric field distribution established by the potential differential between the two electrodes, as described by Equation (6). The charge is calculated by the integration of ***E*** in the conductor contour.

As expected, the highest |E| is found at the area enclosed between the gap and the electrode thickness since this is the region where the electrodes are the closest to each other, i.e., the highest electric potential density. As reported in [25], the maximum ***E*** penetration length in the liquid under test from the electrodes plane occurs at about λ/2 on an interdigital sensing capacitor, i.e., x=λ/2+h in Figure 10 or 368 µm without the SU-8 layer, where |E| averages $1.1819\times 10^{-6}\;V/m$ along the X direction. This means that the sensor is not sensitive beyond this distance. Therefore, considering this maximum penetration length, the total liquid volume required by the transducer to effectively make a permittivity measurement is 1.054 nL for IDEAs 1 and 2 and 1.274 nL for IDEA 3, and it also should be considered that the sample must be in contact with the transducer for at least 126 ms to allow the calculation algorithm to take the corresponding measurements, process the data, and transfer the calculated values through BLE.

**Figure 11:**
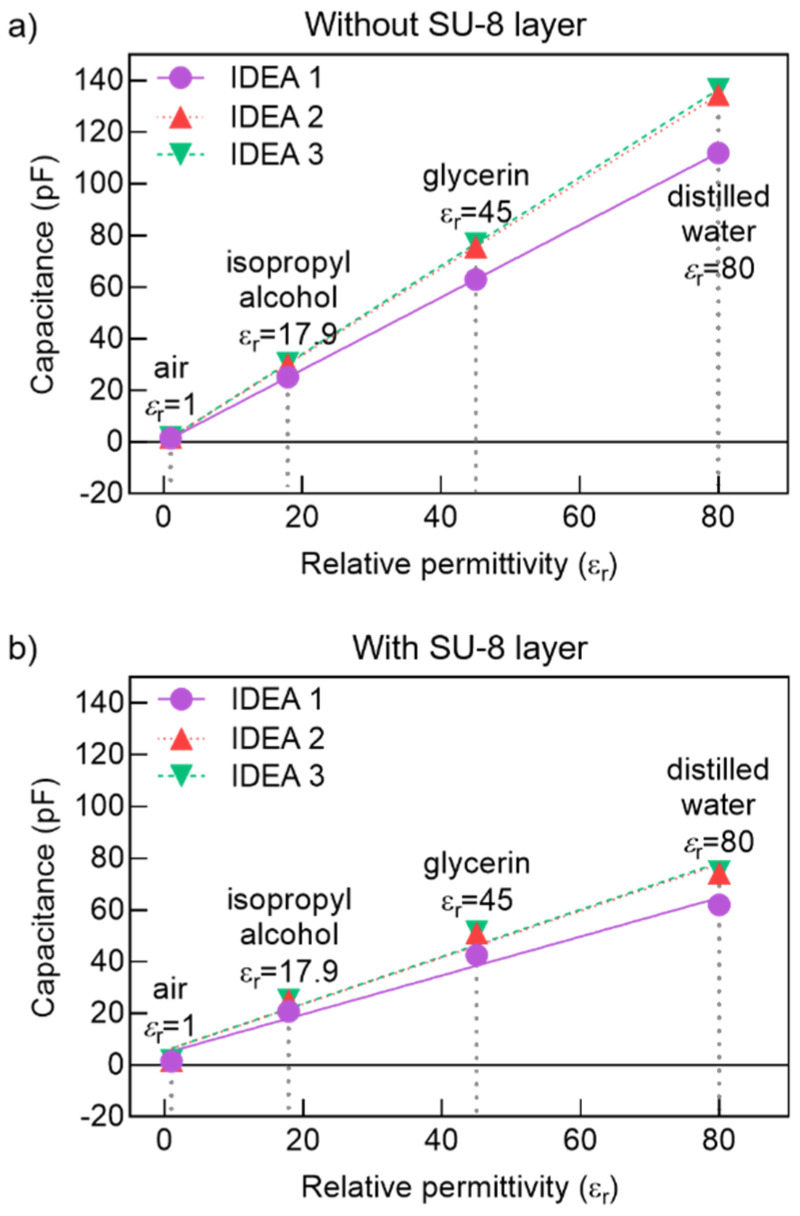
Capacitance simulation results: (**a**) without SU-8 layer and (**b**) with SU-8 layer.

The simulation results for the capacitance of the three IDEA design realizations in the four liquid samples are shown in Figure 11, which makes it evident how the presence of the SU-8 layer reduces the total capacitance of the transducer.

### 3.3. Theoretical Sensitivity Analysis

**Figure 12:**
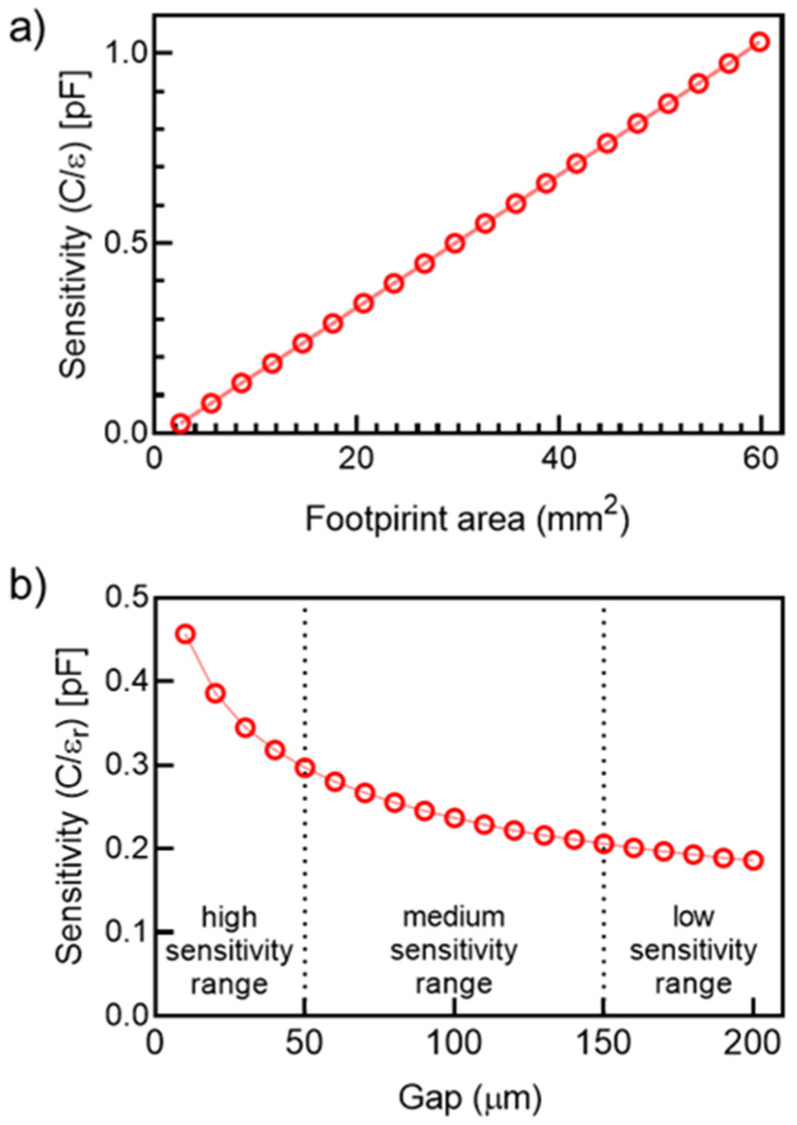
Effects of geometrical parameters of the IDEA on the sensitivity of the transducer: (**a**) effect of the footprint area of the electrodes and (**b**) effect of the gap between electrode fingers.

Figure 12a shows the sensitivity variation, i.e., the slope of the $C_{p}/\varepsilon_{r}$ curve, with respect to changes in the footprint area of the IDEA. In this analysis $\varepsilon_{r}$ = 80 (distilled water), g = 100 µm, l = 4.3 mm, and w = 250 µm, and the number of fingers was varied from N = 4 to 42 in increments of 2. The total footprint area can be approximated as $A_{t}=N\times l\left(w+g\right)$. The linear sensitivity increase with respect to the footprint area of the transducer is due to the stepped effective surface increments of the sensing area due to the addition of electrode pairs.

By fixing the values for $\varepsilon_{r}$ = 80, N = 10, l = 4.3 mm, and w = 250 µm and by varying the gap, which strongly depends on the resolution of the microfabrication process, from g = 10 µm to 200 µm by increments of 10 µm, a decrease in the gap between the fingers considerably increases the sensitivity of the electrodes without significantly increasing the footprint area of the transducer, as shown in Figure 12b.

Since the gap between the electrodes increases while the rest of the geometric variables remain constant, the footprint area linearly increases as a consequence. However, gaps in the range of g ≤ 50 µm have a higher impact on the overall sensitivity than for the cases of g > 50 µm. Considering an electrode width of 250, the high sensitivity range (HSR) for this device was found to be for electrode width/gap aspect ratios of 20 ≥ w:g ≥ 5 since the minimum gap that is achievable is around 12 µm due to the focused laser beam diameter of the laser ablation system.

### 3.4. Experimental Tests

**Figure 13:**
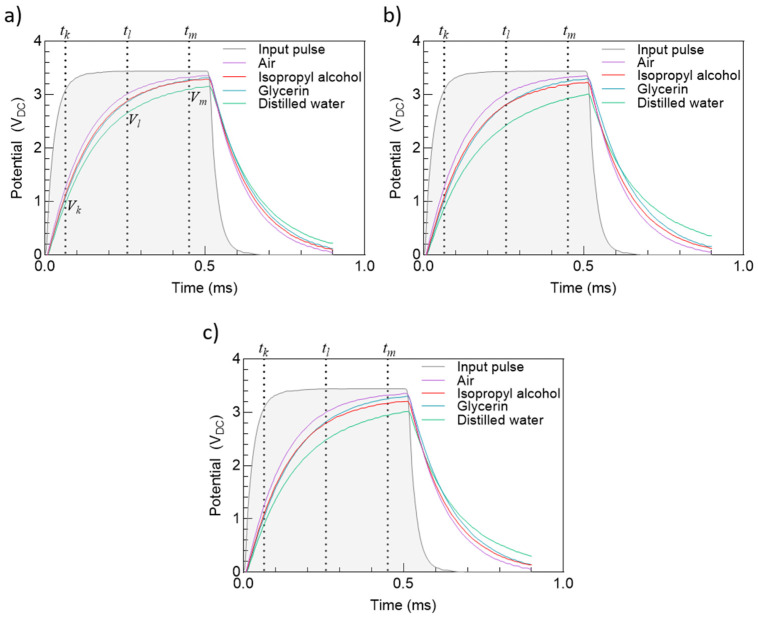
Transducers response to input pulse for different testing fluids: (**a**) IDEA 1, (**b**) IDEA 2, and (**c**) IDEA 3.

The input pulse and the charge and discharge cycles of IDEAs 1, 2, and 3 are shown in Figure 13. Sampling times $t_{k}$, $t_{l}$, and $t_{m}$ indicate measurement points for the voltage values $V_{k}$, $V_{l}$, and $V_{m}$, respectively, which are later used by the MCU to estimate the transducer impedance. To verify the reproducibility of the results, two different devices with the same feature sizes were used for each testing liquid, and the plots represent the mean of the two responses for each device and test fluid.

The transducer response to air is particularly similar in all three cases. This is because air, which has the smallest permittivity value, can be considered to be the path with the highest impedance from the transducer point of view, thus a minimal current flowing through it, and consequently, the transducer reaches the maximum output voltage possible (~3.3 V).

Interestingly, our measurements show that a transition occurs on the measurements between isopropyl alcohol and glycerin as the charging cycle evolves. At $t_{k}$, measurements for IDEA 1 averaged 1.1 V, 2.84 V, and 3.24 V for isopropyl alcohol, and 1.04 V, 2.84 V, and 3.28 V for glycerin. As expected, for $t_{k}$, isopropyl alcohol shows a higher potential than glycerin, by a difference of 0.06 V, due to a lower permittivity value. However, at $t_{l}$, this difference is canceled out to finally reverse at tm by 0.44 V. This effect is consistently observed for IDEAs 2 and 3, with small variations on the potential different at the three sampling times. Finally, measurements for distilled water show the lowest values for all three transductors.

The effect of the sensitivity of the transducer with respect to the footprints area was also analyzed using the three aforementioned transductor geometries. The experimental tests were conducted using the same four testing fluids: air, isopropyl alcohol, glycerin, and distilled water. The capacitance determined from theoretical calculations and experiments is summarized in Table 3. For simplification purposes, the SU-8 layer covering the structures is not considered in the theoretical calculations. A linearity analysis based on the experimental results shows that the transducers presented an average offset of 133 pF, which is a system constant that considers the substrate capacitance and output capacitance of the wave generator or the microcontroller. The system constant was measured by running the algorithm without the transducer connected in an early calibration stage.

On average, IDEA 2 showed the smallest RPE (14%), meaning that IDEA 2 follows the closest to what can be considered the ideal behavior of a theoretical transducer, which is consistent since it is the one with the highest number of fingers N = 24.

**Figure 14:**
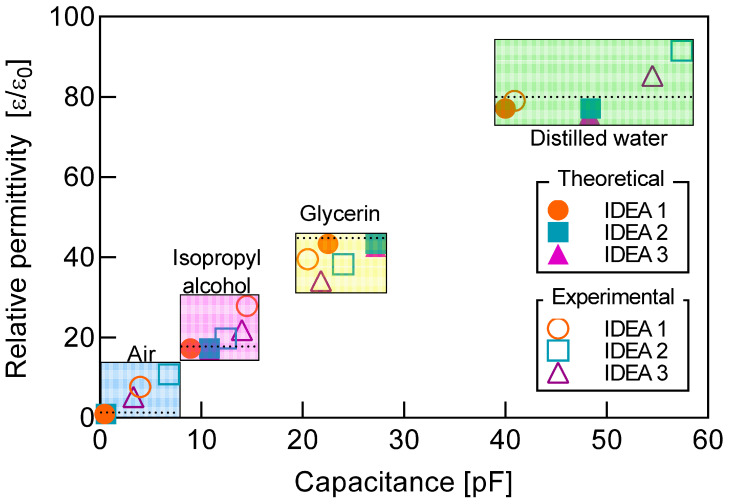
Relative permittivity estimations from experimental capacitance measurements and theoretical calculations for the three IDEAs 1, 2, and 3. Dotted line indicates the standard value for each testing fluid.

From capacitance measurements, relative permittivity can be calculated by reversing the process followed from Equations (1)–(5). In this sense, relative permittivity values calculated from both the experimental measurements and the theoretical calculations are presented in Figure 14.

As expected, permittivity estimations from theoretical capacitance values are located closer to the corresponding standard permittivity value of each testing fluid in almost all cases, while permittivity estimations from experimental measurements are found relatively farther away from this standard value. However, as shown in Figure 14, for all four testing fluids, relative permittivity from both the theoretical and experimental inputs can be enclosed into a narrow range without overlapping, which suggests that for fluids with relative permittivities that are sufficiently spread, the low-cost fabrication process used in this work is suitable for the development of transducers with a high enough differentiation rate. Furthermore, for IDEA 1, which has the overall smallest RPE of all three transducers, the permittivity range could be narrowed even further, thus possibly reducing the permittivity difference among testing fluids.

Both the theoretical and experimental values confirm that the transducers with the highest footprint areas have the highest sensitivities, as shown in Table 4.

A least square fit allows the experimental regression line for the relative permittivity to be obtained as

(7) $$\varepsilon_{r}=\frac{\partial \varepsilon_{r}}{\partial C_{p}}C_{p},$$

where $\partial \varepsilon_{r}/\partial C_{p}≅1.6965\pm 0.2028$, and $C_{p}\;$ is in pF.

## 4. Conclusions

The use of a PCB-based IDEA fabricated by laser ablation allowed the reduction of the transducer footprint while maintaining its sensitivity within an adequate value for the targeted applications. This technique overcame the lack of precision of traditional PCB manufacturing techniques, e.g., chemical etching, while reducing the fabrication time and cost, compared to photolithography, at the prototyping stage. The transducer consisted of interdigitated electrodes that fulfill the characteristics of sensitivity, portability, and scalability for mobile and non-invasive liquid recognition applications, where the dielectric constant must be differentiated.

The system incorporates a previously reported method to analytically determine three independent components of the impedance of a transducer by applying a single square-wave voltage and by measuring the resulting current intensity at only three different selected times. However, this method was implemented into the PSoC CY8C424LQI for the first time, which provides IoT functionality to the sensor node by not only adding on-chip signal processing and signal generation, but also BLE communication capabilities. Hence, the use of an external component was avoided and was reduced to a microcontroller and its internal components.

BLE was used to report transducer changes to external observers. This protocol allowed a battery efficient transmission while only sending data when needed. The protocol overhead was short; hence, the size of the package is kept as small as possible.

## Figures and Tables

**Table 1:** Geometric features of the three fabricated IDEAs.

Geometric Feature	IDEA 1	IDEA 2	IDEA 3
Finger width (w)	250 µm	250 µm	250 µm
Gap between fingers (g)	100 µm	100 µm	100 µm
Finger length (l)	4.3 mm	4.3 mm	5.2 mm
Finger count (N)	20	24	20
Footprint area (A)	30.1 mm^2^	36.1 mm^2^	36.4 mm^2^

**Table 2:** Geometrical characterization of the PCB-MEMS device fabricated from laser ablation.

Geometric Feature	CAD Design	PCB Device Mean (SD)	RPE
w [µm]	250	235.40 (9.7)	6.201%
l [mm]	4.3	4.36 (12.7)	1.578%
g [µm]	100	112.13 (9.5)	10.814%
A [mm^2^]	30.1	30.4 (1.7)	0.878%

**Table 3:** Theoretical and experimental capacitance, pF.

Transducer	Air		Isopropyl Alcohol		Glycerin		Distilled Water	
	Theoretical	Experimental	Theoretical	Experimental	Theoretical	Experimental	Theoretical	Experimental
IDEA 1	0.49	3.98	8.94	14.5	22.48	20.5	39.97	40.9
IDEA 2	0.60	6.82	10.82	12.4	27.22	24.0	48.39	57.4
IDEA 3	0.60	3.32	10.81	14.0	27.19	21.8	48.34	54.5

**Table 4:** Sensitivity relative percentage error between theoretical calculations and experimental results.

Transducer	Footprint Area, mm^2^	Sensitivity, pF/*ε_r_*		
		Theoretical	Experimental	RPE
IDEA 1	30.1	0.5186	0.5181	0.10%
IDEA 2	36.1	0.6274	0.6272	0.04%
IDEA 3	36.4	0.6382	0.6390	0.12%

## Data Availability

Not applicable.
